# Through Hills and Valleys

**DOI:** 10.4269/ajtmh.22-0709

**Published:** 2023-02-06

**Authors:** Sanya Thomas

**Affiliations:** Division of Infectious Diseases, Boston Children’s Hospital and Harvard Medical School, Boston, Massachusetts

As the day came to a close on December 31, 2016, I was preparing to retire for the night and reflect on the year gone by. I was posted in a rural secondary care hospital in the Vellore district of Tamil Nadu in India as part of my Community Health rotation during my internship at Christian Medical College (CMC). My co-intern had taken off to celebrate New Year’s Day with her friends in the main hospital campus and I was alone in the interns’ quarters. It was a cold and quiet night. The emergency room (ER) was empty and our patients in the wards were stable.

Just when I thought I would have a peaceful night, my beeper rang, and I was called to the ER. A middle-aged man had come with breathing difficulty that had worsened over the past couple of hours and showed no signs of improvement with oxygen supplementation. I assessed him quickly and made a diagnosis of probable acute respiratory failure. I knew this was a complicated case, so I paged a senior resident physician for help.

It was soon apparent that the patient was going into shock, probably due to sepsis, and I quickly secured an intravenous line for fluid resuscitation. We decided to transfer him to the main, tertiary-level CMC hospital. We called our ambulance and informed the ER at CMC about the transfer. The resident physician asked me to go along as the patient’s condition could deteriorate during the 30-minute journey.

Sure enough, his blood pressure and oxygen saturation dropped en route. The man’s wife started crying and screaming, and I was praying for strength as this was the first time I was faced with such a medical situation. I quickly consoled the wife and asked her to cooperate as I tried to stabilize her husband, which was extremely hard to do single-handedly in an ambulance rushing at the fastest possible speed, with intermittent bumps and breaks encountered due to poor village roads.

I tried my best to keep him alive by adjusting the fluids and oxygen, but he crashed and lost his pulse. I quickly started chest compressions and asked the man’s wife to place the bag-mask ventilation unit next to me. I switched to bagging after a compression cycle and asked the ambulance driver to drive faster.

As I was on the third CPR cycle, the man’s vital signs showed improvement, and we were almost reaching the hospital. I stopped CPR and adjusted the fluids and oxygen.

I made a quick call to the CMC ER, explained the situation, and informed them that we would need him to be triaged quickly to the resuscitation room. We rushed him inside the resuscitation room as we reached the main ER, and the physicians continued the management of the patient while I gave a handover with his history and presentation. Because the patient and his wife couldn’t afford expensive treatment, I also requested the ER team to consider offering them a concession given their financial situation.

It was almost 2 am as I stepped out of the ER and called the ambulance driver to head back. A lady came running to me and hugged me while tears kept rolling down her cheeks. She kept thanking me with folded hands for saving her husband’s life. I reassured her she was in good hands and that her husband would be taken care of well at CMC. I requested the ambulance driver to wait for a few minutes and took the lady to the coffee shop located next to the ER. I bought her a snack and a cup of tea and asked her to wait in the ER as she was the only bystander with the patient.

As we were driving back, I rolled down the window on my side and looked out in the dark silently. The driver told me I was a good doctor and that I managed the situation well. At that moment, I realized I had started the New Year with saving a life and thanked God for blessing me with this privilege. I was exhausted but the experience was highly rewarding. I followed up on the status of the patient’s condition in the coming days and was happy that he was stable and recovering.

I hope this story highlights the importance of excelling in primary care and the challenges faced by hospitals and caregivers in low- and middle-income countries, particularly in remote and resource-limited settings. It also underscores the urgent need for translating innovative ideas to benefit practice rendered at primary and secondary care centers. The burden became especially high during the past 2 years as we battled the COVID-19 pandemic, and I personally know how hard it has been at such hospitals located in remote parts of India. The high numbers of outpatients and inpatients overwhelm the doctors serving at CMC under normal circumstances, and the pandemic aggravated this situation manyfold. Nevertheless, they served tirelessly and responded to the needs that surfaced with the evolving situation.

Saving a life with minimal resources in a rural area is a skill every doctor must master. The reality in these settings is starkly different from the highly equipped facilities in developed countries, but mastering lifesaving skills with basic resources becomes critical for that one life you as a doctor might be able to save if faced with an emergency.

